# Muscle reoxygenation is slower after higher cycling intensity, and is faster and more reliable in locomotor than in accessory muscle sites

**DOI:** 10.3389/fphys.2024.1449384

**Published:** 2024-08-14

**Authors:** Jem I. Arnold, Assaf Yogev, Hannah Nelson, Martijn van Hooff, Michael S. Koehle

**Affiliations:** ^1^ School of Kinesiology, The University of British Columbia, Vancouver, BC, Canada; ^2^ Department of Pathology and Laboratory Medicine, The University of British Columbia, Vancouver, BC, Canada; ^3^ Department of Sports and Exercise, Máxima Medical Centre, Veldhoven, Netherlands; ^4^ Department of Biomedical Physiology and Kinesiology, Faculty of Science, Simon Fraser University, Burnaby, BC, Canada; ^5^ Division of Sport and Exercise Medicine, Faculty of Medicine, The University of British Columbia, Vancouver, BC, Canada

**Keywords:** near-infrared spectroscopy, muscle oxygenation, muscle oxygen uptake, wearable, exercise testing, repeatability, re-oxygenation, recovery

## Abstract

**Introduction:**

Wearable near-infrared spectroscopy (NIRS) can be used during dynamic exercise to reflect the balance of muscle oxygen delivery and uptake. This study describes the behaviour and reliability of postexercise reoxygenation with NIRS as a function of exercise intensity at four muscle sites during an incremental cycling test. We discuss physiological components of faster and slower reoxygenation kinetics in the context of sport science and clinical applications. We hypothesised that reoxygenation would be slower at higher intensity, and that locomotor muscles would be faster than accessory muscles. We quantified test-retest reliability and agreement for each site.

**Methods:**

Twenty-one trained cyclists performed two trials of an incremental cycling protocol with 5-min work stages and 1-min rest between stages. NIRS was recorded from the locomotor vastus lateralis and rectus femoris muscles, and accessory lumbar paraspinal and lateral deltoid muscles. Reoxygenation time course was analysed as the half-recovery time (HRT) from the end of work to half of the peak reoxygenation amplitude during rest. Coefficient of variability (CV) between participants, standard error of the measurement (SEM) within participants, and intraclass correlation coefficient (ICC) for test-retest reliability were evaluated at 50%, 75%, and 100% peak workloads. A linear mixed-effects model was used to compare differences between workloads and muscle sites.

**Results:**

HRT was slower with increasing workload in the VL, RF, and PS, but not DL. VL had the fastest reoxygenation (lowest HRT) across muscle sites at all workloads (HRT = 8, 12, 17 s at 50%, 75%, 100% workload, respectively). VL also had the greatest reliability and agreement. HRT was sequentially slower between muscle sites in the order of VL < RF < PS < DL, and reliability was lower than for the VL.

**Discussion:**

This study highlights the potential for using wearable NIRS on multiple muscle sites during exercise. Reoxygenation kinetics differ between local muscle sites with increasing intensity. Moderate-to-good reliability in the VL support its increasing use in sport science and clinical applications. Lower reliability in other muscle sites suggest they are not appropriate to be used alone, but may add information when combined to better reflect systemic intensity and fatigue during exercise at different intensities.

## 1 Introduction

Near-infrared spectroscopy (NIRS) is a promising non-invasive, wearable technology that evaluates the balance of oxygen (O_2_) delivery and O_2_ uptake in local tissue during exercise in real time. Commercially available wearable NIRS devices have become more compact, powerful, and accessible in recent years with increasing adoption in sport science and clinical applications (Cornelis et al., 2021; Perrey et al., 2024). Wearable NIRS records the ratio of oxygenated and deoxygenated haemoglobin and myoglobin (oxy- and deoxy[HbMb], respectively), and reports a percent value of muscle oxygen saturation (SmO_2_). SmO_2_ represents the relative O_2_ saturation of Hb and Mb binding sites within the NIRS field of view. Technical details of NIRS can be found elsewhere (Barstow, 2019; Hamaoka and McCully, 2019).

Despite being reported on a 0%–100% scale, wearable SmO_2_ is a relatively scaled measurement with large differences in percent values observed between individuals and between different NIRS devices (McManus et al., 2018). SmO_2_ from two common wearable NIRS devices (Moxy monitor, Fortiori Design LLC and Portamon, Artinis Medical Systems) have been observed with test-retest agreement of approximately ±5–10 %SmO_2_, standard deviation between individuals of ±5–25 %SmO_2_, and mean bias between devices of ±30–45 %SmO_2_ during different exercise tasks (Crum et al., 2017; McManus et al., 2018; van Hooff et al., 2022b; Yogev et al., 2023; Skotzke et al., 2024). Because of this limitation in interpreting ‘absolute’ SmO_2_ values, NIRS research often observes how SmO_2_ changes over time or as a function of exercise intensity (Kirby et al., 2021; Perrey et al., 2024).

The direction and rate of change of SmO_2_ can tell us about the dynamic matching of muscle O_2_ delivery (m $\dot{Q}$ O_2_) and O_2_ uptake (m $\dot{V}$ O_2_) (Hamaoka and McCully, 2019; Kirby et al., 2021). NIRS signals are sensitive to tissue composition, e.g., adipose tissue thickness and cutaneous blood flow, which tend to elevate SmO_2_, reduce deoxygenation during exercise (Niemeijer et al., 2017; McManus et al., 2018; Barstow, 2019), and consequently reduce reoxygenation slope calculations (Keller et al., 2023). However, the time-course of SmO_2_ resaturation after exercise (reoxygenation kinetics) predominantly reflects metabolic changes in muscle tissue and may help to mitigate these limitations (Hamaoka and McCully, 2019).

During recovery from exercise, the energetic demand of work is reduced and energy from m $\dot{V}$ O_2_ is directed toward restoring the metabolic milieu, i.e., phosphocreatine concentration ([PCr]) and other metabolites (Yoshida and Watari, 1993; McCully et al., 1994; Ryan et al., 2013; Hamaoka and McCully, 2019). As recovery proceeds and demand for m $\dot{V}$ O_2_ decays, blood flow and m $\dot{Q}$ O_2_ also decrease, but at a slower rate (Bangsbo and Hellsten, 1998; Van Beekvelt et al., 2001; Behnke et al., 2009). The resulting mismatch in favour of excess m $\dot{Q}$ O_2_ above m $\dot{V}$ O_2_ is observed as reoxygenation.

In general, faster postexercise reoxygenation implies a combination of: (1) greater excess m $\dot{Q}$ O_2_ relative to m $\dot{V}$ O_2_, (2) slower recovery of m $\dot{Q}$ O_2_, and (3) faster recovery of m $\dot{V}$ O_2_ (Bangsbo and Hellsten, 1998; Ichimura et al., 2006; Hamaoka and McCully, 2019). However, higher exercise intensity is associated with greater disruption in muscle metabolic milieu (Yoshida and Watari, 1993) and lower O_2_ availability (i.e., lower SmO_2_), such that m $\dot{V}$ O_2_ recovery may be limited by O_2_ diffusion capacity (Adami and Rossiter, 2018; Pilotto et al., 2022). Therefore, reoxygenation kinetics also may be slower after higher intensity exercise.

Reoxygenation kinetics are of particular interest in sport science and clinical applications. For example, faster reoxygenation is observed after endurance training (Puente-Maestu et al., 2003), and are associated with younger and higher fitness participants (Ichimura et al., 2006; Marwood et al., 2011), and with improved endurance performance across various sporting tasks (Buchheit and Ufland, 2011; Paquette et al., 2020). In clinical evaluation, reoxygenation is consistently shown to be delayed in patients with peripheral vascular disease (Baker et al., 2017; Cornelis et al., 2021), diabetes (Baltrūnas et al., 2021), heart failure (Belardinelli et al., 1997), mitochondrial myopathies (Bravo et al., 2012), and sport-related vascular conditions (van Hooff et al., 2022a; Ritchie et al., 2022). Thus, slower reoxygenation is associated with conditions which impair either m $\dot{Q}$ O_2_ or m $\dot{V}$ O_2_, and often both together.

The majority of sport science and clinical research observes NIRS responses in only one muscle site at a time, most often the quadriceps or calf muscles in the lower limb, and biceps or forearm in the upper extremity (Perrey et al., 2024). During exercise, systemic blood flow and O_2_ delivery is tightly controlled and directed toward the most metabolically active tissues via sympathetic vasoconstriction and locally mediated vasodilation (Laughlin et al., 2012). When NIRS responses are monitored in non-locomotor muscle sites, they appear to demonstrate a reduced metabolic priority (Ogata et al., 2007; Osawa et al., 2017). Therefore, investigating NIRS at multiple muscle sites simultaneously may allow for improved interpretation of how local and systemic processes interact to meet exercise demands (Kirby et al., 2021; Yogev et al., 2022; Perrey et al., 2024).

By observing reoxygenation kinetics across a range of exercise intensities at multiple muscle sites, we hope to gain more insight into how O_2_ delivery is redirected systemically toward tissues with different rates of metabolic activity, and between muscles with different contraction patterns, e.g., postural stabiliser muscles vs. rhythmically contracting locomotor muscles. It will also be useful to understand the reliability and variability between participants for reoxygenation at these less common muscle sites.

### 1.1 Purpose, objective, hypotheses

The primary objective of this paper is to describe the time course of NIRS reoxygenation as a function of exercise intensity during an incremental multi-stage cycling protocol, at multiple locomotor and accessory muscle sites. The secondary objective is to describe between-participant variability, test-retest reliability, and within-participant agreement of reoxygenation time course at these muscle sites.

We hypothesised that (1) reoxygenation would be slower with increasing exercise intensity, and (2) reoxygenation would be faster in locomotor muscles than in accessory muscles.

## 2 Methods

### 2.1 Participants and experimental design

Twenty-one trained cyclists (n = 10 females, n = 11 males; Table 1) volunteered and provided written informed consent to participate in two experimental trials each of an identical incremental multi-stage cycling protocol consisting of 5-min work stages with 1-min rest periods between stages. The workload began at 1.0 W kg^-1^ and increased by 0.5 W kg^-1^ per stage to maximal exercise tolerance. Participants were instructed to maintain the same exercise, diet, and sleep routines for 24 h prior to each visit, and to avoid strenuous exercise for 24 h, and caffeine for 4 h prior. Trials were performed 1 or 2 weeks apart at the same time of day (±1 h). Participants’ body mass and height were recorded at the start of the first visit. Skinfold (SKF) measurements (Harpenden Skinfold Calliper, Baty International, West Sussex, England) were recorded at the placement locations of the NIRS sensors (detailed below). Participants were trained or well-trained and all competitive. This study was approved by the clinical research ethics board of the University of British Columbia and was conducted in accordance with principles established in the Declaration of Helsinki.

**TABLE 1:** Participant characteristics.

Measurement	n = 21
Age (yrs)	29 (8)
Mass (kg)	69.6 (11.3)
Height (cm)	173.6 (11.1)
$\dot{V}$ O_2_peak (ml·min^-1^)	4118 (1045)
$\dot{V}$ O_2_peak (ml·kg^-1^·min^-1^)	58.6 (7.9)
Wpeak (W)	305 (79)
Wpeak (W·kg^-1^)	4.3 (0.6)
Exercise Duration (min)	46.1 (7.4)
VL SKF (mm)	8.0 (5.0–15)
RF SKF (mm)	10.0 (8.0–17)
DL SKF (mm)	11.0 (8.0–13)

Values displayed as mean (SD), except skinfolds displayed as median (Q_1_-Q_3_). n, number of participants. $\dot{V}$ O_2_peak: peak systemic oxygen uptake. Wpeak, peak workload. SKF, skinfold thickness. VL, vastus lateralis. RF, rectus femoris. DL, deltoid.

Experimental design for this study has been reported previously (Yogev et al., 2023). Briefly, cycling exercise was performed on the participants’ own bike mounted to an electronically controlled trainer (Tacx NEO 2T, Garmin International Inc., Olathe, KS, United States) at a self-selected cadence between 80–100 rpm, matched between both trials. Expired metabolic gases were recorded by an open-circuit mixing chamber analyser (TrueOne 2400, ParvoMedics Inc., Sandy, UT, United States). $\dot{V}$ O_2_peak was determined as the highest 30-s average oxygen uptake measurement attained during either of the two trials. Peak workload (Wpeak) was considered the highest completed workload proportional to the total completed duration for the final stage from either of the two trials. In cases where the athlete was not able to reach the same power in their second trial as their first trial, the higher power output was considered Wpeak.

### 2.2 NIRS instrumentation

Muscle oxygenation was recorded with four wearable NIRS sensors (Moxy Monitor, Fortiori Design LLC., Hutchinson, MN, United States) positioned on the vastus lateralis (VL), rectus femoris (RF), lumbar paraspinal (PS), and lateral deltoid (DL) muscles. Placement was on the muscle bellies of the VL at ⅓ the distance from the patella to greater trochanter; the RF at ½ the distance from the patella to anterior superior iliac spine; the PS lateral to the L3 vertebra on the erector spinae; and the DL at ½ the distance from the acromion to the deltoid tuberosity on the humerus. NIRS sensors were positioned with the athlete standing and secured with adhesive tape and a plastic light shield to minimise signal interference from ambient light and movement. All measurements were taken from the right side.

The Moxy monitor is a wearable NIRS sensor which can resolve SmO_2_ on a 0%–100% scale and an arbitrarily scaled concentration of total[HbMb] (Feldmann et al., 2019) from a single LED source and two detectors at 12.5 and 25 mm inter-optode separation, giving a maximum insonation depth of approximately 12.5 mm. SmO_2_ is resolved across the two optode pairs. Technical considerations of the Moxy device are described elsewhere (Crum et al., 2017; McManus et al., 2018; Feldmann et al., 2019).

### 2.3 Data analysis

Data processing and statistical analysis were performed in R version 4.3.3 (R Core Team, 2021) with the packages *lme4* 1.1–35.1 (Bates et al., 2015), *lmerTest* 3.1–3 (Kuznetsova et al., 2017), *emmeans* 1.10.0 (Lenth et al., 2023), and *SimplyAgree* 0.2.0 (Caldwell, 2022). SmO_2_ was analysed as the primary NIRS signal (McManus et al., 2018). SmO_2_ was recorded at 0.5 Hz and smoothed with a 5-s symmetrical moving average to 1 Hz as per manufacturer recommended settings.

**FIGURE 1:**
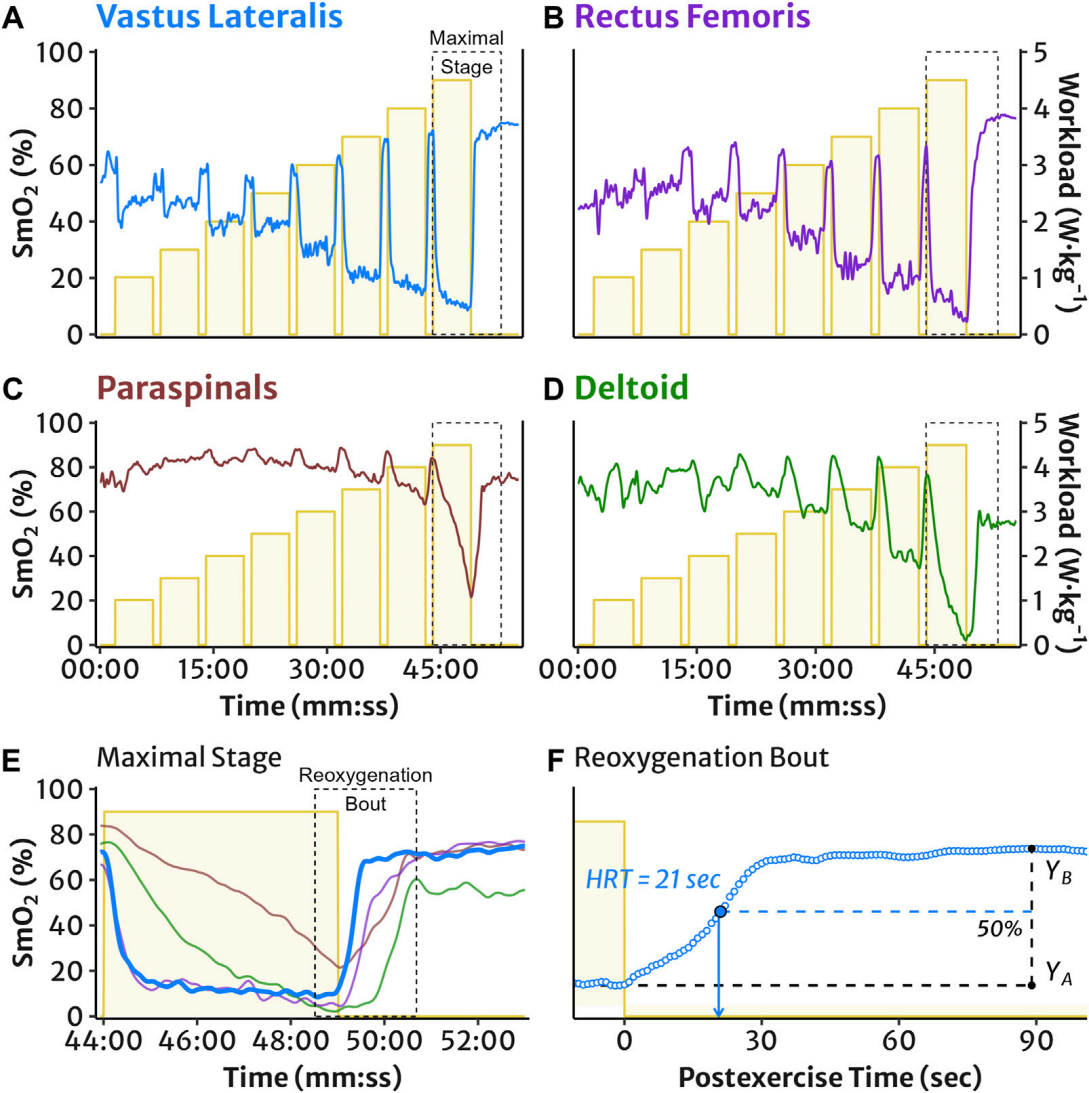
Representative example of the incremental multi-stage cycling protocol and NIRS muscle oxygen saturation (SmO_2_) measured in **(A)** vastus lateralis, **(B)** rectus femoris, **(C)** lumbar paraspinals, and **(D)** lateral deltoid muscles. The maximal work stage is outlined and expanded in panel **(E)**: SmO_2_ demonstrates typical deoxygenation during an exercise interval, followed by postexercise reoxygenation. The maximal reoxygenation bout is outlined and expanded for the VL in panel **(F)**: representative example of VL SmO_2_ reoxygenation half-recovery time (HRT), calculated as the time required to recover half of the total reoxygenation amplitude, i.e. 50% of the difference between the mean SmO_2_ value at the end of work (Y_A_) and the peak recovery SmO_2_ value (Y_B_).

Reoxygenation bouts were evaluated from SmO_2_ during rest periods after each work stage. The mean SmO_2_ value during the final 30 s of the work interval represented the starting value for reoxygenation. The peak SmO_2_ value observed during the rest period represented the ending value. This peak was defined as the first SmO_2_ value with no higher subsequent peaks within 30 s during a recovery time window, which was defined separately for the locomotor and accessory muscles. For locomotor muscle sites (VL and RF), this recovery window was to the start of the subsequent work stage. For accessory muscle sites (PS and DL), this window was to 60 s after the start of the subsequent work stage. The recovery window after the final work stage was 4 min for all muscles. The total reoxygenation amplitude was the difference between the peak and end-work mean SmO_2_ values. Reoxygenation time course was quantified as the half-recovery time (HRT), calculated as the time required to recover half of the total SmO_2_ amplitude (see representative schematic in Figure 1F).

Of the 504 total reoxygenation bouts recorded across 21 participants, two trials, three target work stages, and four muscle sites, there were 43 bouts (8.5%) with missing data where HRT could not be calculated. In two trials, the NIRS recording software failed after the final work stage and reoxygenation was lost for all sites at 100% workload. The NIRS device was not secured well enough at the PS in five trials and the DL in two trials, resulting in unusable data due to noise from light and/or movement. The device failed to record entirely for two trials in the RF, one trial each at the PS and DL, and in one individual reoxygenation bout each at the PS and DL.

We also analysed reoxygenation kinetics with a monoexponential function, however over 25% of the reoxygenation bouts did not conform to a specific phenomenological curve, evaluated by a coefficient of determination pseudo-R^2^ ≥0.85 and physiologically plausible mean response time between zero and the time of the peak SmO_2_ value. Therefore, we chose to only discuss the non-parametric HRT estimation of reoxygenation time. Please see Limitations section for further discussion, and Supplementary Material for results of the monoexponential kinetics analysis.

### 2.4 Between-participant variability and test-retest reliability

Between-participant variability was determined for HRT at work stages corresponding to 50%, 75%, and 100% Wpeak for each of the four muscle sites. This was quantified for absolute variability as the group-level median and interquartile range (Q_1_-Q_3_ or IQR) and for relative variability as the coefficient of variation (CV) between participants. Relative reliability was quantified as intraclass correlation coefficient (ICC_2,1_) and interpreted as poor (<0.5), moderate (0.5–0.75), good (0.75–0.9), and excellent (>0.9) (Koo and Li, 2016). Absolute test-retest reliability and within-participant agreement was quantified as the standard error of the measurement (SEM) and minimal detectable change (MDC), in original units of seconds (Atkinson and Nevill, 1998; Weir, 2005). Within-participant CV was also calculated.

### 2.5 Statistical analysis

Data are displayed as mean (SD) where normally distributed, and as median (Q_1_-Q_3_) where non-normally distributed, unless indicated otherwise. Alpha for all comparisons was set to 0.05. To compare reoxygenation across exercise intensity and between muscle sites, a linear mixed-effects model was used to analyse HRT with fixed effects for workload (%Wpeak with a continuous quadratic spline) and muscle site (VL, RF, PS, DL). Random effects of participant were included for the model slope and intercept. Trial was initially included as an additional fixed effect, however the final chosen model excluding trial was preferred based on the Akaike Information Criterion (AIC) and Bayesian information criterion (BIC). Pairwise *post hoc* estimated marginal means were contrasted between workloads and muscle sites at 50%, 75%, and 100% Wpeak using the Tukey method to correct for multiple comparisons. Model residuals were examined visually from Q-Q and other residual plots to check for normality and heteroscedasticity.

## 3 Results

The incremental multi-stage cycling protocol and HRT measurement of SmO_2_ reoxygenation are shown in Figure 1. Descriptive reoxygenation data and group-level coefficients of variation at 50%, 75%, and 100% workloads are reported in Table 2.

**TABLE 2:** Reoxygenation descriptive data at work stages corresponding to 50%, 75%, and 100% peak workload.

		Workload		
Muscle Site	HRT (sec)	51 (3)%	75 (3)%	100 (1)%
Vastus Lateralis	n	21	21	21
	Median (Q₁-Q₃)	8 (6–10)	12 (10–13)	17 (16–20)
	CV (%)	30	29	17
Rectus Femoris	n	21	21	21
	Median (Q₁-Q₃)	18 (14–26)	20 (16–30)	32 (22–49)
	CV (%)	50	45	47
Paraspinals	n	19	19	18
	Median (Q₁-Q₃)	26 (17–32)	26 (24–30)	39 (33–52)
	CV (%)	45	29	40
Deltoid	n	21	21	21
	Median (Q₁-Q₃)	36 (30–49)	38 (30–42)	42 (36–51)
	CV (%)	38	33	37

Workloads displayed as mean (SD) %Wpeak. HRT: half-recovery time. n: number of participants with available reoxygenation data. CV: coefficient of variation between participants.

### 3.1 Comparison of reoxygenation with increasing exercise intensity

**FIGURE 2:**
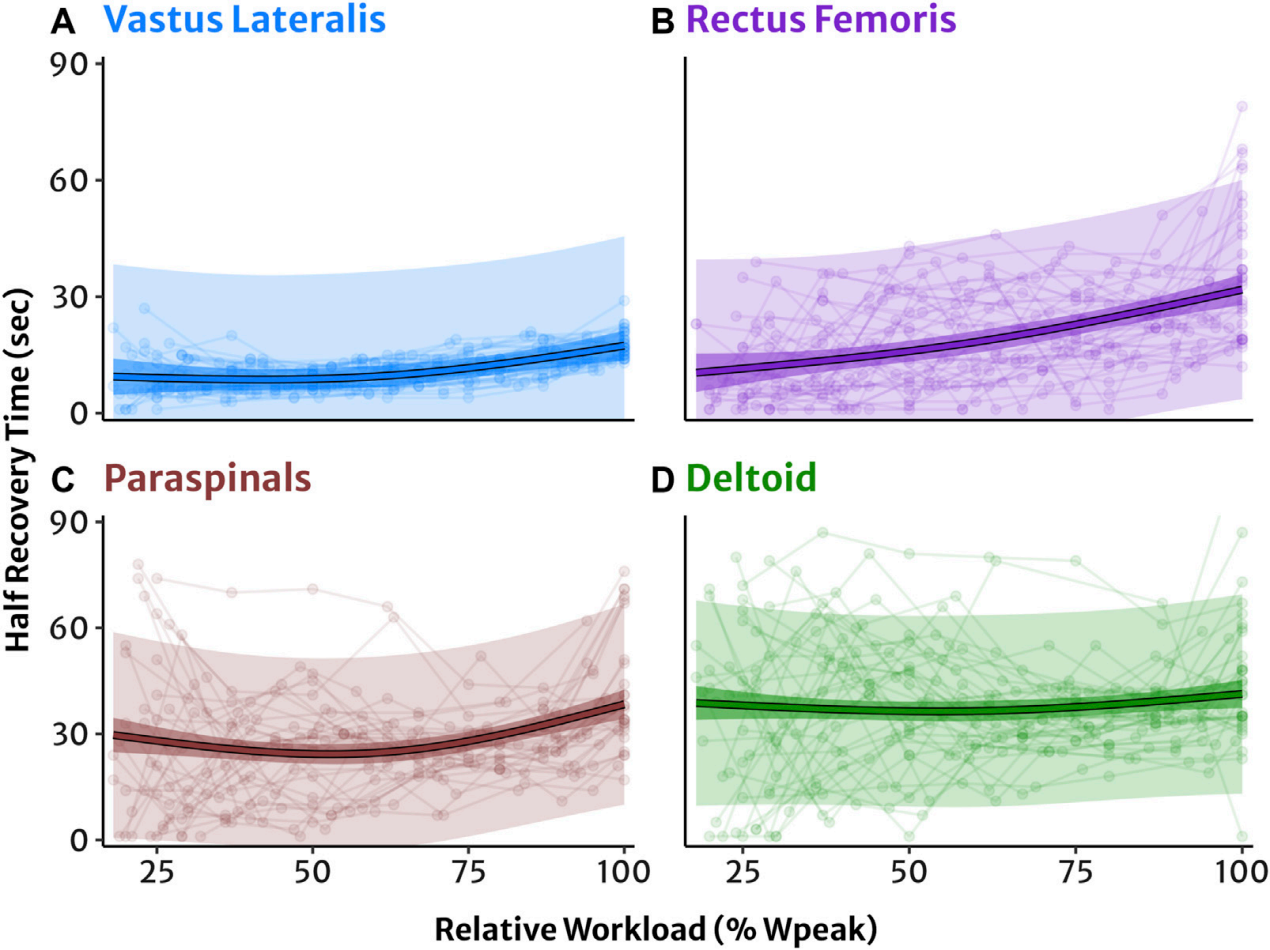
NIRS reoxygenation in four muscle sites modelled as a function of exercise intensity relative to each participant’s 100% peak workload. **(A)** Vastus lateralis, **(B)** rectus femoris, **(C)** lumbar paraspinals, and **(D)** lateral deltoid muscles. Half-recovery time (HRT) in seconds with individual datapoints (dots and lines). Predicted trendlines, dark shaded 95% confidence intervals, and light shaded 95% prediction intervals derived from the linear mixed-effects model.

Linear mixed-effects modelling of HRT as a function of exercise intensity in four muscle sites are shown in Figure 2. *Post hoc* comparisons of estimated marginal mean HRT between 50%, 75%, and 100% workloads indicated that reoxygenation was significantly slower sequentially with increasing workload in all muscle sites (all *p* < 0.05) apart from the DL where HRT was not different across workloads (Table 3). See Supplementary Table S1 for full HRT model coefficients.

**TABLE 3:** Estimated marginal contrasts of workloads between reoxygenation at 50%, 75%, and 100% peak workload in each muscle site from the linear mixed-effects model.

Muscle Site	Contrast of Workload (95% CI)		
	75%–50%	100%–50%	100%–75%
Vastus Lateralis	3 (1, 5)*	9 (3, 14)*	6 (2, 10)*
Rectus Femoris	7 (5, 9)*	16 (10, 22)*	9 (5, 13)*
Paraspinals	4 (2, 6)*	14 (8, 20)*	10 (6, 15)*
Deltoid	1 (−1, 3)	5 (−1, 11)	4 (−1, 8)

Values represent the estimate (95% confidence interval) for the pairwise difference in half-recovery time (HRT) in seconds between workloads. Positive values indicate that the higher workload has slower reoxygenation than the lower workload. * significant difference (*P* < 0.05) for the contrast between workloads.

### 3.2 Comparison between locomotor and accessory muscle sites

*Post hoc* comparisons of estimated marginal mean HRT between each muscle site at 50%, 75%, and 100% workloads indicated that reoxygenation was significantly slower sequentially in order of VL < RF < PS < DL (*p* < 0.05) at each workload, except for between the PS and DL which were not different at 100% (Table 4).

**TABLE 4:** Estimated marginal contrasts of muscle sites between reoxygenation at 50%, 75%, and 100% peak workload from the linear mixed-effects model.

Contrast of muscle sites (95% CI)	Workload		
	50%	75%	100%
RF - VL	7 (4, 11)*	11 (8, 15)*	14 (9, 20)*
PS - VL	16 (12, 19)*	16 (13, 20)*	21 (15, 27)*
PS - RF	8 (4, 12)*	5 (2, 9)*	6 (0, 13)*
DL - VL	28 (24, 31)*	26 (22, 29)*	24 (18, 30)*
DL - RF	20 (17, 24)*	15 (11, 18)*	10 (4, 15)*
DL - PS	12 (8, 16)*	10 (6, 13)*	3 (−3, 9)

VL, vastus lateralis; RF, rectus femoris; PS, paraspinals; DL, deltoid; Values represent the estimate (95% confidence interval) for the pairwise difference in half-recovery time (HRT) in seconds between muscle sites. Positive values indicate that the first listed muscle site has slower reoxygenation than the second site.* significant difference (*p* < 0.05) for the contrast between muscle sites.

### 3.3 Between-participant variability and test-retest reliability

**FIGURE 3:**
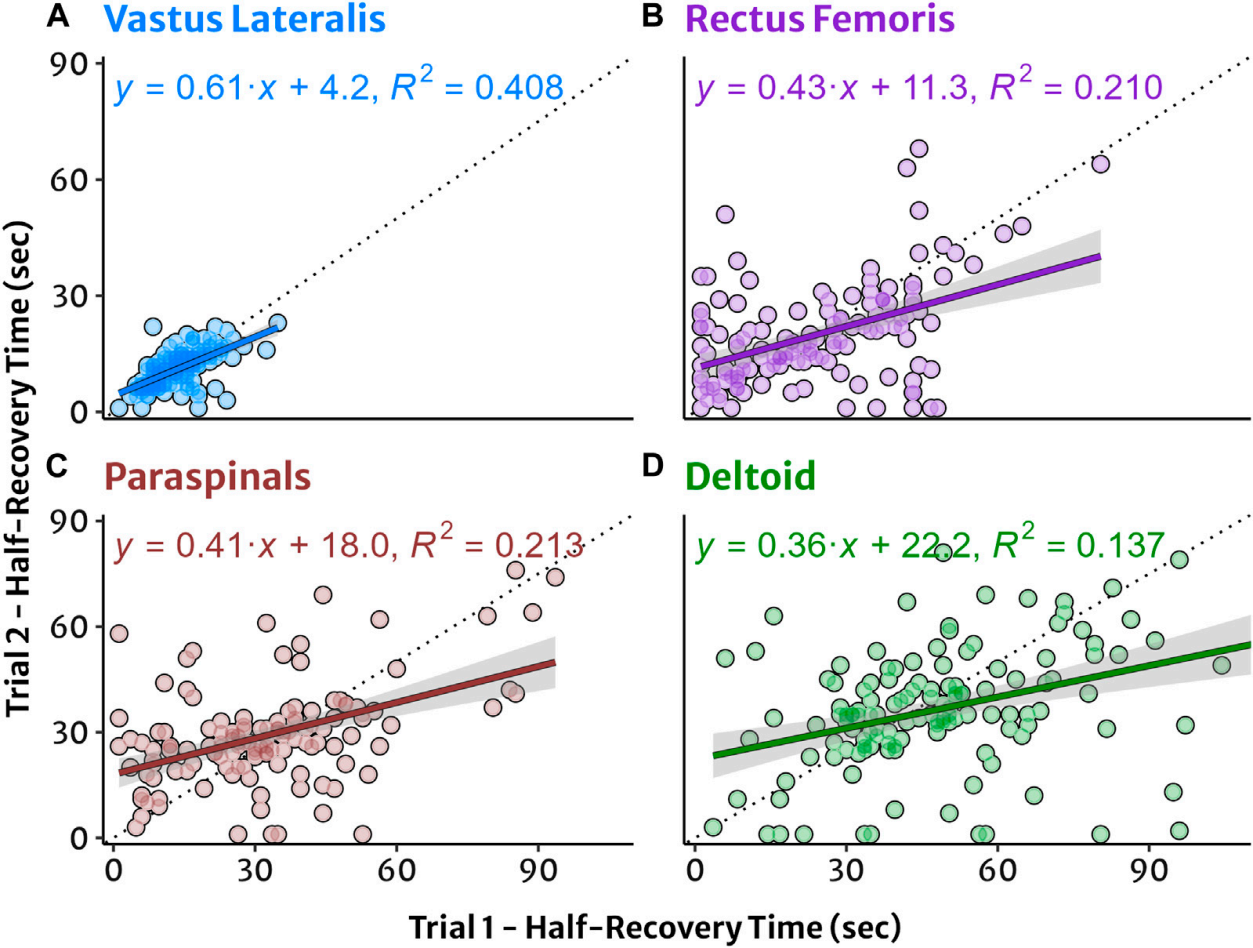
Test‐retest agreement for NIRS reoxygenation in four muscle sites plotted from trial 1 and trial 2. (A) Vastus lateralis, (B) rectus femoris, (C) lumbar paraspinals, and (D) lateral deltoid muscles. Half‐recovery time (HRT) in seconds with individual datapoints. Simple linear regression trendlines and shaded 95% confidence interval for HRT with equation and coefficient of determination (R^2^) provided for descriptive information.

Test-retest agreement for HRT values between trial 1 and 2 are shown in Figure 3. Between-participant variability tended to be greater in absolute (IQR) and relative (CV) terms in sequential order of VL < PS < DL < RF at equivalent workloads (Table 2). Within-participant agreement tended to be lower in absolute terms (SEM, MDC) in the order of VL < RF ≈ PS < DL, but in relative terms (CV) RF had the greatest variability and lowest agreement. This resulted in mostly poor to moderate relative reliability (ICC) estimates, with good reliability observed only in the VL at 50% and 75% workloads. Descriptive data for test-retest reliability and within-participant agreement are reported in Table 5.

**TABLE 5:** Reoxygenation reliability and within-participant agreement at 50%, 75%, and 100% peak workload.

		Workload		
Muscle Site	HRT (sec)	51 (3)%	75 (3)%	99 (2)%
Vastus Lateralis	n	21	21	19
	ICC (95% CI)	0.73 (0.49, 0.87)	0.78 (0.59, 0.89)	0.42 (0.06, 0.69)
	SEM (95% CI)	1 (1, 2)	2 (1, 2)	3 (2, 4)
	MDC	4	5	7
	CV (%)	15	14	15
Rectus Femoris	n	19	19	17
	ICC (95% CI)	0.12 (−0.29, 0.49)	0.39 (0.00, 0.67)	0.68 (0.38, 0.85)
	SEM (95% CI)	12 (9, 18)	9 (7, 13)	10 (7, 15)
	MDC	33	25	27
	CV (%)	61	43	28
Paraspinals	n	17	17	15
	ICC (95% CI)	0.19 (−0.25, 0.56)	0.56 (0.17, 0.79)	0.72 (0.42, 0.88)
	SEM (95% CI)	14 (11, 22)	5 (4, 8)	8 (6, 12)
	MDC	39	15	22
	CV (%)	56	20	20
Deltoid	n	18	17	16
	ICC (95% CI)	0.14 (−0.26, 0.50)	0.36 (−0.05, 0.67)	0.47 (0.08, 0.74)
	SEM (95% CI)	18 (13, 26)	7 (5, 10)	15 (11, 23)
	MDC	49	18	41
	CV (%)	46	19	33

Workloads displayed as mean (SD) %Wpeak. HRT, half-recovery time; n, number of participants with available reoxygenation data; ICC, intraclass correlation coefficient; SEM, standard error of the measurement in seconds; MDC, minimal detectable change in seconds; CV, coefficient of variability for repeated measurements within participants.

## 4 Discussion

This study aimed to describe and compare NIRS reoxygenation in multiple muscle sites, as a function of increasing exercise intensity during an incremental multi-stage cycling protocol with a wearable NIRS device (Moxy monitor). The main findings were that reoxygenation was slower (HRT was greater) with increasing exercise intensity in locomotor VL and RF muscles, and fastest in the primary locomotor VL compared to the RF and accessory PS and DL muscle sites. Descriptively, the between-participant variability tended to be lowest and test-retest reliability tended to be highest for the VL compared to other muscle sites, although variability was not statistically compared.

### 4.1 Locomotor reoxygenation across exercise intensity

Reoxygenation was slower with increasing workload in locomotor muscle sites (VL and RF) across the observed intensity range. This was consistent with our first hypothesis. However, reoxygenation in accessory muscles PS and DL was more variable overall and tended to be less sensitive to changes in workload.

Zuccarelli et al. (2020) recorded reoxygenation kinetics of deoxy[HbMb] in the VL after cycling exercise and found that reoxygenation was faster after a moderate intensity work bout compared to either a heavy intensity work bout or a maximal incremental test. There was no difference between the heavy and maximal intensity reoxygenation, whereas we observed differences between the analogous 75% and 100% workloads (Table 3). This discrepancy could be related to cycling protocol, where the moderate, heavy, and maximal incremental work bouts were performed on different days in the previous study, whereas we observed them during a single incremental protocol in the present experiment.

Previous experiments have shown equivocal findings regarding muscle recovery after exercise at different intensities: The kinetics of [PCr] resynthesis appear to be unchanged with exercise intensity, while inorganic phosphate ([P_i_]) recovers more slowly after higher intensity exercise (Yoshida and Watari, 1993; Rossiter et al., 2002). m $\dot{V}$ O_2_ recovery is related to the overall resynthesis of the metabolic milieu, and m $\dot{V}$ O_2_ measured via arterial/venous blood sampling is observed to recover more slowly after higher intensity exercise related to a greater metabolite disruption (Yoshida and Watari, 1993; Krustrup et al., 2009). Recent experiments have used a non-invasive method with NIRS and repeated arterial occlusions to measure m $\dot{V}$ O_2_ recovery after exercise, which is well correlated with [PCr] resynthesis (Ryan et al., 2013; Adami and Rossiter, 2018). This method has also shown slower m $\dot{V}$ O_2_ recovery in the VL after higher intensity knee extension (Tripp et al., 2023) and cycling exercise (Zuccarelli et al., 2020).

The experiment from Zuccarelli et al. (2020) simultaneously monitored m $\dot{V}$ O_2_ recovery with repeated occlusions in one leg, and ‘free-flow’ (i.e., un-occluded) deoxy[HbMb] reoxygenation in the other leg after cycling exercise. Deoxy[HbMb] reoxygenation kinetics were similar to m $\dot{V}$ O_2_ recovery after moderate intensity cycling, but were further delayed after both heavy and maximal exercise. This may be related to lower O_2_ availability after higher intensity exercise which is briefly insufficient to maximally activate m $\dot{V}$ O_2_ (Adami and Rossiter, 2018; Pilotto et al., 2022). Beever et al. (2020) and others have observed that m $\dot{V}$ O_2_ measurements taken immediately after muscle contractions occasionally exhibit a delay before the start of systematic recovery. This suggests that reoxygenation can be briefly rate limited by O_2_ diffusion (Pilotto et al., 2022), despite higher intensity exercise stimulating elevated m $\dot{Q}$ O_2_ (blood volume and flow) with vasodilation (Bangsbo and Hellsten, 1998; Laughlin et al., 2012). Collectively, these experiments may explain why postexercise SmO_2_ reoxygenation in ‘free-flow’ conditions in the locomotor muscles are progressively delayed with increasing intensity in the present incremental cycling protocol.

Koga et al. (2017) also measured deoxy[HbMb] reoxygenation in the VL and RF after cycling in the heavy intensity domain. They observed that reoxygenation kinetics in the VL were faster than in the RF, consistent with our findings. The mean response time (MRT) of a monoexponential function had mean (SD) values of 29 (10) s in the VL, and 77 (37) s in the RF. These are somewhat slower than the median (IQR) HRT values observed in the present study at the analogous 75% Wpeak, of 12 (3) s and 20 (14) s in the VL and RF, respectively (Table 2). MRT represents the time to recover approximately 63% of the reoxygenation amplitude, whereas HRT is calculated at 50%, and so MRT is expected to be larger. Even accounting for this, the faster reoxygenation observed in the present study could also be explained by participant fitness level: we evaluated trained and competitive cyclists, while the previous study investigated untrained participants (Koga et al., 2017).

Reoxygenation kinetics have been shown to be faster in individuals with higher fitness (Ichimura et al., 2006) and after endurance training (Puente-Maestu et al., 2003) as a consequence of enhanced adaptations toward better and faster matching of m $\dot{Q}$ O_2_ and m $\dot{V}$ O_2_ (Buchheit and Ufland, 2011; Beever et al., 2020; Paquette et al., 2020). m $\dot{V}$ O_2_ recovery with the repeated occlusion method is also faster with higher $\dot{V}$ O_2_max (Beever et al., 2020), enhanced mitochondrial content (Tripp et al., 2023), and improved endurance performance (Batterson et al., 2020). Increased mitochondrial protein content and respiratory capacity (Puente-Maestu et al., 2003; Tripp et al., 2023) increases the rate at which m $\dot{V}$ O_2_ can restore muscle metabolic milieu (e.g., [PCr] and [P_i_]), insofar as m $\dot{Q}$ O_2_ is sufficient to saturate O_2_ diffusion from capillaries to mitochondria (Adami and Rossiter, 2018; Pilotto et al., 2022). Therefore, peripheral adaptations toward increased capillarisation and improved vasodilatory function, and centrally improved blood volume, cardiac output, and sympathetically mediated vascular function will help to drive both increased convective and diffusive O_2_ delivery (Buchheit and Ufland, 2011; Laughlin et al., 2012; Hellsten and Nyberg, 2015).

We did not correlate our findings to athlete fitness level, and so future research should clarify not only whether reoxygenation kinetics are enhanced, but whether the reliability of reoxygenation measurements also changes with training and fitness. Additionally, reoxygenation in non-locomotor muscle may interact differently with fitness (e.g., kinetics may be slower or unchanged with intensity) as blood flow is prioritised toward locomotor muscle sites and other critical tissues (Ogata et al., 2007; Laughlin et al., 2012; Osawa et al., 2017).

### 4.2 Comparison between muscle sites

Reoxygenation was fastest in the VL muscle and sequentially slower in the RF, PS, and DL, consistent with our second hypothesis. The VL is more involved than the RF during cycling, as demonstrated by muscle contraction activity measured with electromyography (EMG) and deoxygenation with NIRS (Hug and Dorel, 2009; Chin et al., 2011; Iannetta et al., 2017). The biarticular RF is described as having more of a secondary role through the pedal cycle to accelerate the pedal over top dead centre, while the uniarticular VL strongly activates during the pushing phase (Hug and Dorel, 2009). The RF is less efficient and has greater variability in power production between athletes than the VL (Hug and Dorel, 2009; Blake and Wakeling, 2015), which may explain the higher variability observed in the present study. It is difficult to isolate m $\dot{V}$ O_2_ and m $\dot{Q}$ O_2_ components of the NIRS signal in each quadricep muscle independently, but Okushima et al. (2020) has speculated that the RF may be more dependent on substrate metabolism than on O_2_ extraction, related to the different activation pattern. Slower reoxygenation in the RF compared to VL at cycling workloads above 50% Wpeak in the present experiment are consistent with this hypothesis. Thus, the different recruitment and metabolic patterns in the RF compared to VL may contribute to both the slower reoxygenation and the higher variability observed in the RF.

Osawa et al. (2017) observed slower reoxygenation in the upper extremity biceps brachii (BB) compared to the VL after 30 s high intensity cycling intervals, consistent with the present results in the DL. They also observed wider variability between participants at the BB compared to VL. Slower reoxygenation and wider variability could be explained by differences in recruitment of the upper extremity for stabilisation during cycling, particularly at higher workloads (Ozyener et al., 2012). However, deoxygenation is observed in the upper extremity even when the arm is supported and m $\dot{V}$ O_2_ is not increased for stabilisation (Shiroishi et al., 2010). This deoxygenation is thought to be related to a systemic redirection of blood flow and m $\dot{Q}$ O_2_ away from less-involved muscles via sympathetic vasoconstriction toward more metabolically active locomotor muscles and critical tissues such as the brain, heart, and respiratory muscles (Ogata et al., 2007; Laughlin et al., 2012; Osawa et al., 2017). For this reason, we expected the upper extremity to show further delays in reoxygenation at higher intensity. However, the DL was not sensitive to increasing intensity with any consistency among the participants (Table 3). The variability in the DL could be related to autonomic differences influencing systemic sympathetic vasoconstriction (Ogata et al., 2007; Laughlin et al., 2012; Osawa et al., 2017), and local differences in thermoregulatory blood flow to the skin of the upper extremity (Simmons et al., 2011; Messere and Roatta, 2013). Given the smaller muscle mass in the upper extremity, skinfold differences may have also contributed to greater variability (discussed further in limitations).

Data are sparse on the NIRS responses of the spinal erector muscles during cycling. Kell and Bhambhani (2007) recorded NIRS in the lumbar paraspinals during a horizontal isometric stabilisation task to maximal tolerance and recovery. They observed reoxygenation with a mean (SD) HRT of 35 (22) s. This is comparable to our median (IQR) of 39 (19) s after 100% workload. EMG activity of the spinal erector muscles increases in proportion to cycling workload, as they contribute to stabilisation and efficient energy transfer to pedalling (Muyor et al., 2022). While the contractile forces likely differ between an isometric hold and rhythmic pedalling, the similar reoxygenation time course observed after these tasks may indicate a common matching of m $\dot{Q}$ O_2_ and m $\dot{V}$ O_2_ at the respective maximal task tolerance. Given that low back pain is a common overuse symptom reported by cyclists (Clarsen et al., 2010) and that recruitment of the spinal erectors changes with low back pain (Muyor et al., 2022), it is interesting to speculate whether observing NIRS on the paraspinals during cycling may have value for performance, bike fit, and/or clinical applications. However, in a similar experiment to the one above, Langenfeld et al. (2022) found that reoxygenation in the PS was not reliable enough to use for clinical evaluation of patients with low back pain (ICC = −0.225-0.252). We found moderate reliability in the PS after 75% and 100% cycling workloads, and so it remains to be seen whether this parameter would be sensitive to performance or clinical evaluation in a cycling context.

The sequence in the time course of reoxygenation observed across the four sites investigated, representing primary and secondary locomotor, postural stabiliser, and upper extremity muscles, points toward the prioritisation of more metabolically active tissues and systemic redirection of blood flow and O_2_ delivery to meet those metabolic demands. The variability observed in the non-locomotor muscle sites could simply be biological noise, or it could help to understand how systemic responses arise from an aggregation of heterogenous local responses.

### 4.3 Test-retest reliability and within-participant agreement

Relative reliability was evaluated as ICC and represents the proportion of measurement error attributable to, e.g., biological variability and instrument error, compared to the variability observed between individuals (Atkinson and Nevill, 1998; Weir, 2005). ICC was poor-to-good for the four muscle sites investigated at 50%, 75%, and 100% workloads (Table 5). While the RF and PS had moderate reliability at higher workloads, the VL had good reliability at lower workloads and poor reliability after 100% Wpeak. The DL reliability was poor at all workloads. Poor ICC suggests that not only was there high variability in HRT between participants, but the variability in repeated measurements between the two trials was even greater. This is also demonstrated by the high SEM and CV values (low within-participant agreement) for repeated measurements (Table 5). SEM represents a range of values an individual might see with repeated testing day-to-day, while MDC represents a minimum change that would need to be seen to confidently attribute that change to a particular intervention rather than chance alone (Atkinson and Nevill, 1998; Weir, 2005). Given that the participants in this study represented a heterogenous group of mixed sex, fitness levels, tissue composition, and training histories, these values may be larger than those expected for a more homogenous group, such as athletes at the same competitive level.

Reoxygenation was most consistent between trials in the VL, with SEM of 1–3 s and MDC 4–7 s across cycling workloads. While MDC does not imply a minimal clinically important difference between participants, a lower measurement error in general will improve the ability to detect meaningful differences between clinical groups or in athletic performances (Atkinson and Nevill, 1998). Previous studies in healthy participants have found mean differences between groups for reoxygenation kinetics of 7–16 s related to age and fitness (Ichimura et al., 2006; Marwood et al., 2011) and 12–20 s after endurance training (Puente-Maestu et al., 2003). Even larger differences are observed in clinical evaluation, e.g., 7–30 s between healthy cyclists and cyclists with a sport-related vascular condition limiting blood flow to the legs (van Hooff et al., 2022a; van Hooff et al., 2022b). Differences of approximately 15 s are observed in patients with mitochondrial myopathy compared to healthy controls (Bravo et al., 2012), and within-participant changes of 20 s are observed in patients with peripheral vascular disease before vs. after exercise training (Baker et al., 2017). These clinically important differences fall well outside the MDC found in the present study, which is encouraging for the use of reoxygenation in the VL to detect meaningful differences between performance groups and after training interventions.

van Hooff et al. (2022b) reported reoxygenation kinetics in the VL after two trials of maximal incremental cycling in healthy cyclists and in patients with blood flow limitations in the iliac arteries (FLIA). They observed mean (SD) HRT values of approximately 22 (13) s for healthy athletes and 30 (12) s for patients with FLIA. For healthy athletes, this is comparable to the median (IQR) HRT of 17 (4) s observed in the present study (Table 2). They also reported test-retest absolute bias (95% limits of agreement) of −1.2 (18.7) s for healthy athletes and 3.4 (20.3) s for patients, with ICC of 0.59 and 0.64 for each group, respectively. These ICC values are higher than that observed in the present study after 100% workload, however the limits of agreement are also considerably larger than the 4–7 s presently observed for MDC (Table 5). Higher ICC despite larger within-participant variability as observed by van Hooff et al. (2022b) can be explained by relatively greater between-participant variability, compared to the present findings. These discrepancies could be related to the different protocol and NIRS device used, or to a wider heterogeneity between participants in the previous study, which recruited recreational, competitive, and professional athletes. On the other hand, the similar mean HRT values observed between these experiments suggests that different NIRS devices may return similar reoxygenation kinetics, whereas the same two devices report very different ‘absolute’ %SmO_2_ values (McManus et al., 2018). However, a direct comparison of reoxygenation kinetics between these devices would be valuable to understand the potential sources of signal variability.

### 4.4 Applications of reoxygenation kinetics

We now understand that postexercise reoxygenation is dependent on both metabolic O_2_ exchange (i.e., m $\dot{Q}$ O_2_ and m $\dot{V}$ O_2_ matching), and on blood volume perfusion and O_2_ diffusion which contributes to further delays at higher intensity. The repeated occlusion method for evaluating m $\dot{V}$ O_2_ recovery (Adami and Rossiter, 2018; Beever et al., 2020) is a more rigorous protocol for isolating muscle oxygen uptake and oxidative capacity. However, in a field-testing environment where occlusions are not possible, free-flow SmO_2_ reoxygenation may give a holistic view of the ability for muscle to reperfuse and resynthesise the local metabolic milieu with no additional methodological burden (e.g., equipment or protocol constraints) (Buchheit and Ufland, 2011; Paquette et al., 2020).

The present findings suggest that postexercise reoxygenation in the VL has satisfactory test-retest reliability to be used for monitoring longitudinal training effects and to detect cross-sectional differences between groups. Recent work in trained cyclists with FLIA has found that post-maximal exercise reoxygenation in the VL is discriminative of blood flow and O_2_ delivery limitations between FLIA patients and healthy athletes, and even more subtle differences between patients’ affected and unaffected limbs (van Hooff et al., 2022a). Thus, VL reoxygenation appears to be sensitive enough to detect pathological local imbalances in O_2_ delivery and O_2_ uptake. We observed higher reliability in the VL at 50% and 75% Wpeak than at 100% workload, raising the possibility that submaximal reoxygenation may be even more discriminative or predictive of individual performance differences than after maximal exercise. Interestingly, Beckitt et al. (2012) observed that after endurance training, patients with peripheral vascular disease had faster reoxygenation in the gastrocnemius after submaximal exercise, but not after the now higher maximal exercise level attained post-training. This was suggested to reflect an increased work capacity with no change in oxygen debt, facilitated by improved matching of m $\dot{Q}$ O_2_ to m $\dot{V}$ O_2_ throughout exercise.

Reliability was low for the RF, PS, and DL suggesting that monitoring these muscle sites alone is not appropriate for detecting longitudinal changes. However, combining individual observations of multiple muscle sites may reveal more about systemic changes not captured by the primary locomotor muscle. For example, there are individual differences expected in SmO_2_ values between left and right legs (Skotzke et al., 2024), and yet during exercise onset, averaging the NIRS kinetics from left and right VL corresponded to systemic $\dot{V}$ O_2_ kinetics better than either leg alone (Reinpõld and Rannama, 2023). It is possible that aggregating responses across muscle sites could provide information about systemic responses useful for monitoring acute training load and fatigue. However, the utility and application of NIRS measurements at accessory muscle sites remains speculative.

### 4.5 Limitations

Adipose tissue thickness and SKF influence SmO_2_ values and reoxygenation rates (slopes) (Niemeijer et al., 2017; McManus et al., 2018; Keller et al., 2023), however the time course of NIRS responses are expected to be less sensitive to superficial tissue layers (Hamaoka and McCully, 2019). SKF is recommended to be no greater than ½ the inter-optode distance for NIRS research (Barstow, 2019), which was 25 mm (SKF cut-off of 12.5 mm) in the present study. However, no exclusions were made based on skinfolds to prioritise ecological validity for a heterogenous population of male and female competitive cyclists. Seven participants (7 F) had SKF >12.5 mm in the VL, nine (8 F) in the RF, and seven (5 F) in the DL (SKF at the PS was not recorded). The study from van Hooff et al. (2022b) excluded participants with SKF >15 mm at the VL, and yet they still observed greater variability than we observed in the present findings. We investigated the effects of SKF by including it as an additional fixed factor in the linear mixed-effects model, however SKF explained only an additional 5% of the total variance (conditional *R* ^2^ = 0.581 vs. 0.529 for the chosen model), therefore it was not included in the chosen model. However, future research should more thoroughly investigate the effects of SKF on reoxygenation kinetics, which may have interactions with sex and exercise intensity.

In the present study, the DL, and to a lesser extent PS reoxygenation were visibly delayed compared to the locomotor muscle sites, such that reoxygenation continued beyond the 60-s rest period between work intervals. Whereas the locomotor muscles reached their peak values within the passive rest periods. For this reason, the recovery window used to model reoxygenation was extended for the two accessory muscles to include the first 60 s during the subsequent work interval, where PS and DL were observed to eventually reach their peak reoxygenation values and subsequently begin to deoxygenate. At the start of the subsequent work stage, restarting pedalling and the ‘muscle pump’ effect of rhythmic contractions may have increased venous return from the lower extremity and facilitated further reoxygenation in the accessory muscles compared to passive rest (Laughlin et al., 2012). This may have contributed to the greater variability observed in the accessory muscles between and within participants (Tables 2, 5).

Finally, reoxygenation kinetics are commonly described by the MRT of a monoexponential function, however over 25% of our data did not conform to monoexponential kinetics and would have been lost if we restricted our analysis to this method. Therefore, estimating reoxygenation non-parametrically with half-recovery time was a compromise to compare across our heterogenous dataset from different muscles and exercise intensities. Whereas MRT describes and can be used to predict the shape of the response, HRT is a descriptive estimate of reoxygenation and may not represent the same underlying physiological phenomena. Therefore, caution must be advised when comparing HRT values, for example, between different muscle sites. However, the results were not meaningfully different from either HRT or MRT (available in the Supplementary Material), therefore we believe our interpretations are consistent with either calculation method. An advantage of HRT is that it can be easily manually calculated by practitioners without advanced processing or coding. However, we encourage practitioners and researchers to carefully consider the limitations of using any single parameter to describe the complex physiological phenomena occurring during reoxygenation. Future research should develop empirically derived and mechanistically robust equations to describe muscle oxygenation behaviours during exercise onset and recovery.

### 4.6 Conclusion

Our main objective was to describe the behaviour and the variability of reoxygenation with wearable NIRS in four muscle sites (locomotor VL and RF, accessory PS and DL) as exercise intensity increased during an incremental multi-stage cycling protocol. We observed that reoxygenation was generally slower with increasing workload in VL, RF, and PS, but that DL was not sensitive to exercise intensity. The primary locomotor VL muscle tended to have the fastest reoxygenation at all workloads followed sequentially by the more proximal muscle sites in the order VL < RF < PS < DL. Between-participant variability was lowest and within-participant reliability was highest in the VL, whereas the RF showed the highest variability and lowest reliability. Of the four muscles evaluated, only the VL appeared to have sufficiently high test-retest reliability to be used alone for monitoring change with training interventions or for detecting differences between groups. Future research should investigate whether observing NIRS responses across multiple muscle sites together may be useful to help explain day-to-day variations in performance and training responses.

## Data Availability

The raw data supporting the conclusions of this article will be made available by the authors, without undue reservation.
