# Supplementary material for: Muscle reoxygenation is slower after higher cycling intensity, and is faster and more reliable in locomotor than in accessory muscle sites
Source: Front Physiol. 2024 Aug 14;15:1449384. doi: 10.3389/fphys.2024.1449384 (PMC11349675; doi:10.3389/fphys.2024.1449384)
Supplement: Supplementary file 1 [file DataSheet1.docx]

| Supplemental Table 1: Half Recovery Time (HRT) Mixed Effects Model Coefficients | | | | | | | | | |
| --- | --- | --- | --- | --- | --- | --- | --- | --- | --- |
| **effect** | **term** | **estimate** | **std.error** | **statistic** | **df** | **conf.low** | **conf.high** | **p.value** | **p.format** |
| **Fixed Effects** | (Intercept) | 9.451 | 2.370 | 3.988 | 110.9 | 4.755 | 14.147 | < 0.001 | * |
|  | splines::ns(Workload, df = 2)1 | 3.380 | 4.876 | 0.693 | 268.4 | -6.221 | 12.981 | 0.489 |  |
|  | splines::ns(Workload, df = 2)2 | 8.800 | 2.341 | 3.759 | 99.7 | 4.155 | 13.444 | < 0.001 | * |
|  | SiteRF | 0.268 | 2.889 | 0.093 | 1,161.7 | -5.400 | 5.937 | 0.926 |  |
|  | SitePS | 20.151 | 2.843 | 7.088 | 1,160.3 | 14.573 | 25.729 | < 0.001 | * |
|  | SiteDL | 29.083 | 2.813 | 10.337 | 1,157.6 | 23.563 | 34.602 | < 0.001 | * |
|  | splines::ns(Workload, df = 2)1:SiteRF | 18.561 | 6.511 | 2.851 | 1,160.5 | 5.786 | 31.336 | 0.004 | * |
|  | splines::ns(Workload, df = 2)2:SiteRF | 6.503 | 2.851 | 2.281 | 1,158.7 | 0.909 | 12.096 | 0.023 | * |
|  | splines::ns(Workload, df = 2)1:SitePS | -7.221 | 6.448 | -1.120 | 1,159.5 | -19.872 | 5.430 | 0.263 |  |
|  | splines::ns(Workload, df = 2)2:SitePS | 3.754 | 2.916 | 1.287 | 1,166.2 | -1.968 | 9.476 | 0.198 |  |
|  | splines::ns(Workload, df = 2)1:SiteDL | -5.496 | 6.383 | -0.861 | 1,157.3 | -18.020 | 7.028 | 0.389 |  |
|  | splines::ns(Workload, df = 2)2:SiteDL | -5.294 | 2.855 | -1.854 | 1,158.4 | -10.896 | 0.308 | 0.064 |  |
| **Random Effects** | SD (Intercept) | 7.345 |  |  |  |  |  |  |  |
|  | Corr (Intercept): Workload | -0.842 |  |  |  |  |  |  |  |
|  | SD Workload | 9.173 |  |  |  |  |  |  |  |
|  | SD Residual | 12.109 |  |  |  |  |  |  |  |
| **Explained Variance** | Marginal R² | 0.367 |  |  |  |  |  |  |  |
|  | Conditional R² | 0.537 |  |  |  |  |  |  |  |
| Model Formula = HRT ~ splines::ns(Workload, df = 2) + Site + (1 + Workload \| Subject) + splines::ns(Workload, df = 2):Site | | | | | | | | | |
| Marginal R² is the proportion of variance explained by fixed effects. | | | | | | | | | |
| Conditional R² is the proportion of variance explained by fixed and random effects. | | | | | | | | | |

| Supplemental Table 2: Reoxygenation kinetics mean response time (MRT)  descriptive data at work stages corresponding to 50%, 75%, and 100%  peak workload | | | | |
| --- | --- | --- | --- | --- |
|  | | **Workload** | | |
| **Muscle Site** | **MRT (sec)** | **51 (3)%** | **75 (3)%** | **100 (1)%** |
| **Vastus Lateralis** | n | 21 | 21 | 21 |
|  | Median (Q₁-Q₃) | 9 (7-10) | 13 (11-15) | 19 (17-21) |
|  | CV (%) | 29 | 26 | 19 |
| **Rectus Femoris** | n | 10 | 16 | 21 |
|  | Median (Q₁-Q₃) | 15 (11-25) | 22 (18-28) | 42 (34-62) |
|  | CV (%) | 51 | 43 | 43 |
| **Paraspinals** | n | 9 | 15 | 17 |
|  | Median (Q₁-Q₃) | 25 (16-31) | 25 (22-28) | 44 (37-51) |
|  | CV (%) | 35 | 21 | 42 |
| **Deltoid** | n | 17 | 19 | 17 |
|  | Median (Q₁-Q₃) | 38 (25-50) | 32 (29-41) | 47 (39-58) |
|  | CV (%) | 45 | 25 | 36 |

Workloads displayed as mean (SD) %Wpeak. MRT: mean response time. n: number of participants with available reoxygenation data. CV: coefficient of variation between participants.

| Supplemental Table 3: Estimated marginal contrasts of workloads  between reoxygenation kinetics at 50%, 75%, and 100% peak  workload in each muscle site from the linear mixed-effects model | | | |
| --- | --- | --- | --- |
|  | **Contrast of Workload (95% CI)** | | |
| **Muscle Site** | **75% - 50%** | **100% - 50%** | **100% - 75%** |
| **Vastus Lateralis** | 4 (2, 6) * | 10 (4, 15) * | 6 (2, 10) * |
| **Rectus Femoris** | 11 (8, 13) * | 32 (25, 38) * | 21 (16, 26) * |
| **Paraspinals** | 5 (2, 8) * | 23 (16, 30) * | 18 (13, 23) * |
| **Deltoid** | -1 (-4, 2) | 8 (1, 14) * | 8 (4, 13) * |

Values represent the estimate (95% confidence interval) for the pairwise difference in mean response time (MRT) in seconds between workloads. Positive values indicate that the higher workload has slower reoxygenation kinetics than the lower workload. * significant difference (p<0.05) for the contrast between workloads.

| Supplemental Table 4: Estimated marginal contrasts of muscle sites  between reoxygenation kinetics at 50%, 75%, and 100% peak  workload from the linear mixed-effects model | | | |
| --- | --- | --- | --- |
| **Contrast of Muscle Sites (95% CI)** | **Workload** | | |
|  | **50%** | **75%** | **100%** |
| **RF - VL** | 4 (-1, 8) | 10 (6, 14) * | 25 (20, 31) * |
| **PS - VL** | 11 (7, 15) * | 12 (8, 16) * | 24 (19, 30) * |
| **PS - RF** | 7 (2, 13) * | 2 (-3, 7) | -1 (-7, 5) |
| **DL - VL** | 28 (24, 32) * | 23 (19, 26) * | 26 (20, 31) * |
| **DL - RF** | 24 (19, 29) * | 13 (8, 17) * | 0 (-6, 6) |
| **DL - PS** | 17 (12, 21) * | 11 (6, 15) * | 1 (-4, 7) |

VL: vastus lateralis. RF: rectus femoris. PS: paraspinals. DL: deltoid. Values represent the estimate (95% confidence interval) for the pairwise difference in mean response time (MRT) in seconds between muscle sites. Positive values indicate that the first listed muscle site has slower reoxygenation kinetics than the second site. * significant difference (p<0.05) for the contrast between muscle sites.

| Supplemental Table 5: Reoxygenation kinetics mean response time (MRT) reliability  and within-participant agreement at 50%, 75%, and 100% peak workload | | | | |
| --- | --- | --- | --- | --- |
|  | | **Workload** | | |
| **Muscle Site** | **MRT (sec)** | **51 (3)%** | **75 (3)%** | **99 (2)%** |
| **Vastus Lateralis** | n | 18 | 21 | 18 |
|  | ICC (95% CI) | 0.75 (0.50, 0.88) | 0.76 (0.55, 0.88) | 0.51 (0.14, 0.75) |
|  | SEM (95% CI) | 1 (1, 2) | 2 (1, 3) | 3 (2, 5) |
|  | MDC | 4 | 5 | 8 |
|  | CV (%) | 15 | 14 | 16 |
| **Rectus Femoris** | n | 3 | 8 | 10 |
|  | ICC (95% CI) | 0.03 (-2.14, 0.93) | 0.62 (0.03, 0.88) | 0.53 (-0.02, 0.83) |
|  | SEM (95% CI) | 6 (3, 36) | 4 (3, 9) | 15 (11, 28) |
|  | MDC | 16 | 12 | 43 |
|  | CV (%) | 46 | 20 | 37 |
| **Paraspinals** | n | 7 | 8 | 14 |
|  | ICC (95% CI) | 0.67 (0.10, 0.91) | 0.49 (-0.02, 0.82) | 0.63 (0.26, 0.83) |
|  | SEM (95% CI) | 5 (3, 11) | 3 (2, 6) | 13 (9, 21) |
|  | MDC | 14 | 9 | 35 |
|  | CV (%) | 21 | 14 | 28 |
| **Deltoid** | n | 7 | 11 | 12 |
|  | ICC (95% CI) | 0.07 (-0.68, 0.68) | 0.64 (0.24, 0.86) | 0.31 (-0.20, 0.69) |
|  | SEM (95% CI) | 20 (13, 43) | 6 (4, 11) | 17 (12, 29) |
|  | MDC | 55 | 17 | 47 |
|  | CV (%) | 46 | 18 | 37 |

Workloads displayed as mean (SD) %Wpeak. MRT: mean response time. n: number of participants with available reoxygenation data. ICC: intraclass correlation coefficient. SEM: standard error of the measurement in seconds. MDC: minimal detectable change in seconds. CV: coefficient of variability for repeated measurements within participants.

| Supplemental Table 6: Mean Response Time (MRT) Mixed Effects Model Coefficients | | | | | | | | | |
| --- | --- | --- | --- | --- | --- | --- | --- | --- | --- |
| **effect** | **term** | **estimate** | **std.error** | **statistic** | **df** | **conf.low** | **conf.high** | **p.value** | **p.format** |
| **Fixed Effects** | (Intercept) | 6.633 | 3.077 | 2.156 | 178.7 | 0.561 | 12.705 | 0.032 | * |
|  | splines::ns(Workload, df = 2)1 | 11.431 | 6.528 | 1.751 | 246.4 | -1.425 | 24.288 | 0.081 |  |
|  | splines::ns(Workload, df = 2)2 | 11.107 | 2.506 | 4.431 | 48.0 | 6.068 | 16.146 | < 0.001 | * |
|  | SiteRF | 8.326 | 4.621 | 1.802 | 701.6 | -0.746 | 17.399 | 0.072 |  |
|  | SitePS | 25.838 | 4.692 | 5.507 | 714.1 | 16.626 | 35.050 | < 0.001 | * |
|  | SiteDL | 45.591 | 4.025 | 11.328 | 722.3 | 37.690 | 53.492 | < 0.001 | * |
|  | splines::ns(Workload, df = 2)1:SiteRF | 1.953 | 9.728 | 0.201 | 716.6 | -17.145 | 21.052 | 0.841 |  |
|  | splines::ns(Workload, df = 2)2:SiteRF | 21.716 | 3.151 | 6.892 | 723.0 | 15.530 | 27.902 | < 0.001 | * |
|  | splines::ns(Workload, df = 2)1:SitePS | -25.016 | 9.867 | -2.535 | 722.1 | -44.387 | -5.646 | 0.011 | * |
|  | splines::ns(Workload, df = 2)2:SitePS | 9.530 | 3.127 | 3.047 | 724.6 | 3.390 | 15.670 | 0.002 | * |
|  | splines::ns(Workload, df = 2)1:SiteDL | -40.202 | 8.490 | -4.735 | 722.9 | -56.871 | -23.534 | < 0.001 | * |
|  | splines::ns(Workload, df = 2)2:SiteDL | -7.555 | 2.927 | -2.581 | 712.4 | -13.301 | -1.808 | 0.010 | * |
| **Random Effects** | SD (Intercept) | 7.179 |  |  |  |  |  |  |  |
|  | Corr (Intercept):Workload | -0.861 |  |  |  |  |  |  |  |
|  | SD Workload | 12.175 |  |  |  |  |  |  |  |
|  | SD Residual | 10.739 |  |  |  |  |  |  |  |
| **Explained Variance** | Marginal R² | 0.502 |  |  |  |  |  |  |  |
|  | Conditional R² | 0.656 |  |  |  |  |  |  |  |
| Model Formula = MRT ~ splines::ns(Workload, df = 2) + Site + (1 + Workload \| Subject) + splines::ns(Workload, df = 2):Site | | | | | | | | | |
| Marginal R² is the proportion of variance explained by fixed effects. | | | | | | | | | |
| Conditional R² is the proportion of variance explained by fixed and random effects. | | | | | | | | | |


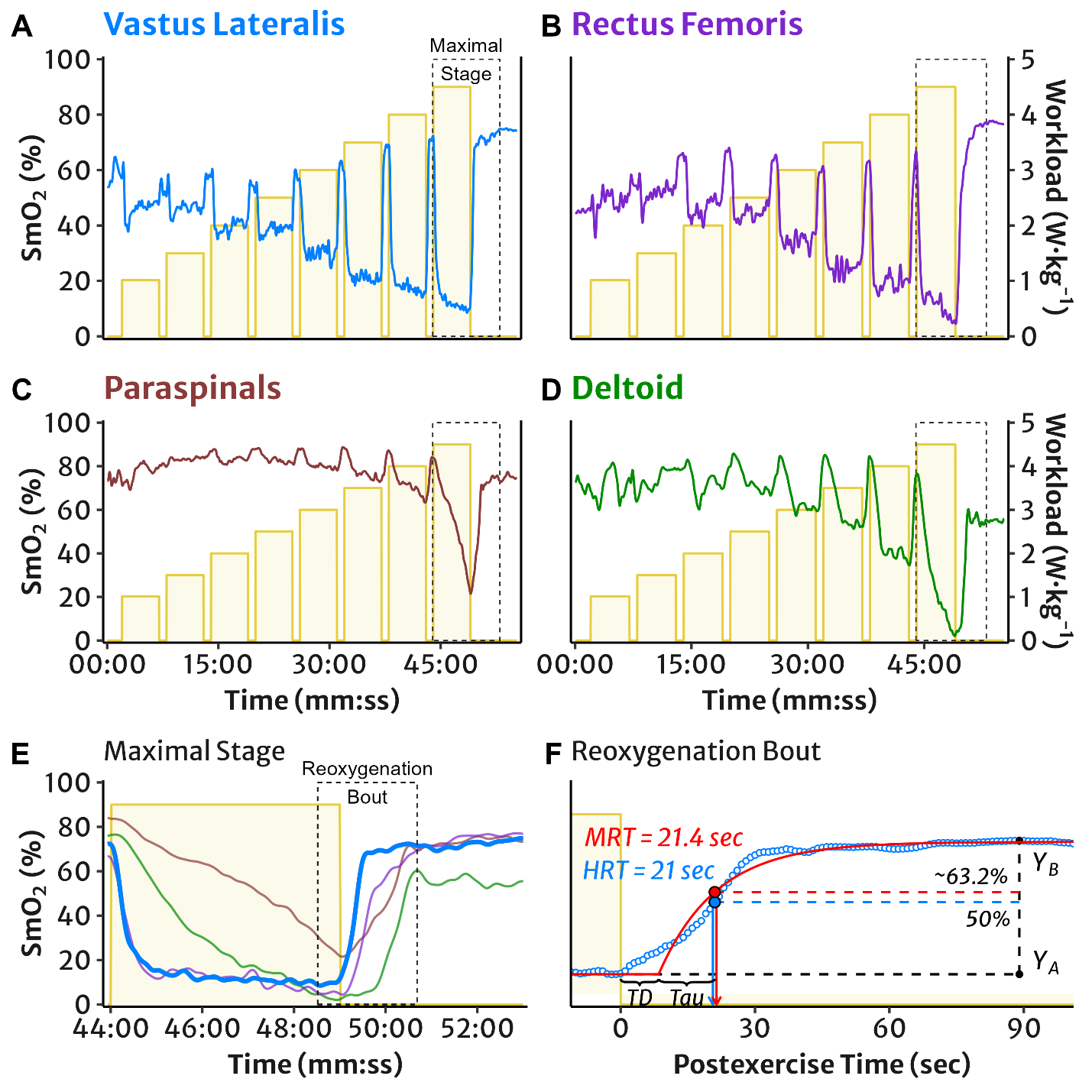


**Supplemental Figure 1** Representative example of the incremental multi-stage cycling protocol and NIRS muscle oxygen saturation (SmO_2_) measured in (A) vastus lateralis, (B) rectus femoris, (C) lumbar paraspinals, and (D) lateral deltoid muscles. The maximal work stage is outlined and expanded in panel (E): SmO_2_ demonstrates typical deoxygenation during an exercise interval, followed by postexercise reoxygenation. Reoxygenation for VL only is outlined and expanded in panel (F): representative example of VL SmO_2_ reoxygenation mean response time (MRT) and half-recovery time (HRT). MRT is the sum of time delay (TD) and time constant (Tau) of a monoexponential function. HRT is calculated as the time required to recover half of the total reoxygenation amplitude, i.e. 50% of the difference between the mean SmO_2_ value at the end of work (Y_A_) and the peak recovery SmO_2_ value (Y_B_).


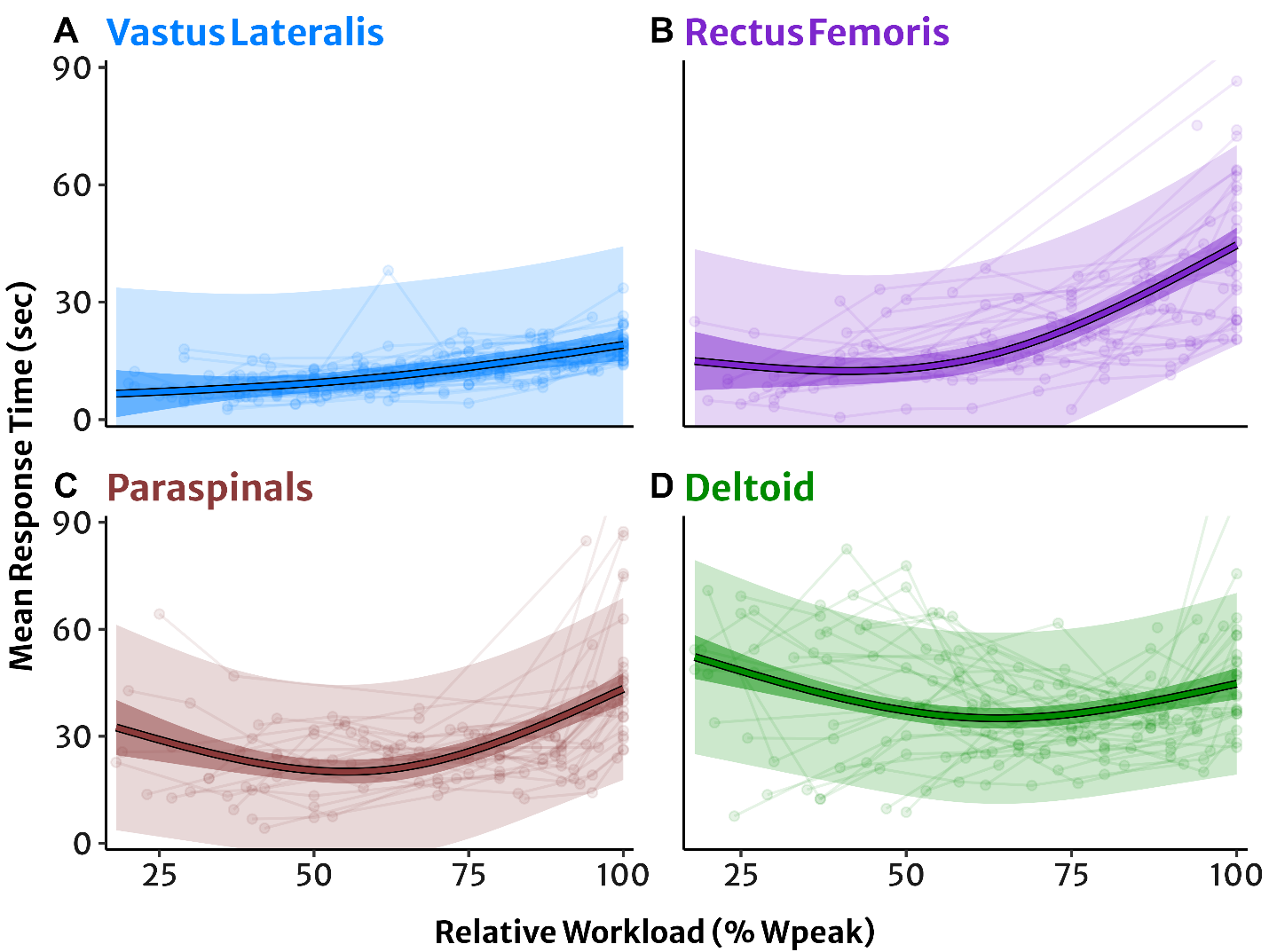


**Supplemental Figure 2** NIRS reoxygenation in four muscle sites modelled as a function of exercise intensity relative to each participant’s 100% peak workload. (A) Vastus lateralis, (B) rectus femoris, (C) lumbar paraspinals, and (D) lateral deltoid muscles. Mean response time (MRT) in seconds with individual datapoints (dots and lines). Predicted trendlines, dark shaded 95% confidence intervals, and light shaded 95% prediction intervals derived from the linear mixed-effects model.
